# One-stage laparoscopic surgery for inspissated bile syndrome: case report and review of surgical techniques

**DOI:** 10.1186/2193-1801-2-648

**Published:** 2013-12-04

**Authors:** Steffen Berger, Susanne Schibli, Enno Stranzinger, Dietmar Cholewa

**Affiliations:** Department of Pediatric Surgery, Inselspital University Hospital, University of Berne, Berne, 3010 Switzerland; Division of Pediatric Gastroenterology, Department of Pediatrics, Inselspital University Hospital, University of Berne, Berne, 3010 Switzerland; Division of Pediatric Radiology, Department of Radiology, Inselspital University Hospital, University of Berne, Berne, 3010 Switzerland

## Abstract

Inspissated bile syndrome in a 6 week old boy was unresponsive to oral ursodesoxycholic acid. Intraoperative cholangiography revealed complete obstruction of the common bile duct. Therefore, the gallbladder fundus was pulled out through a laparoscopy port site and sutured to the fascia. A catheter was positioned into the infundibulum for irrigation with ursodesoxycholic acid. At day 8 complete resolution of the plug and free passage of contrast medium into the duodenum was documented radiologically. The catheter was removed, skin closed spontaneously without a second surgery for closure of the gall bladder.

## Introduction

Neonatal hyperbilirubinemia associated with extrahepatic bile duct dilatation may be caused by inspissated bile. In the absence of metabolic disease or bile duct malformations this inspissation is often preceeded by sludge in the gall bladder e.g. secondary to prolonged parenteral nutrition, sepsis, or hemolysis (Gubernick et al. 1990). The resulting cholestasis may be transient with resolution of the concrements either spontaneously or after medical treatment with ursodesoxycholic acid. In some cases however, medical treatment is unsuccessful and inspissated bile syndrome develops which may require surgical intervention.

We herein report a case of inspissated bile syndrome treated surgically by laparoscopic catheter insertion into the gall bladder for antegrade flushing of the bile ducts.

## Case report

**Figure 1:**
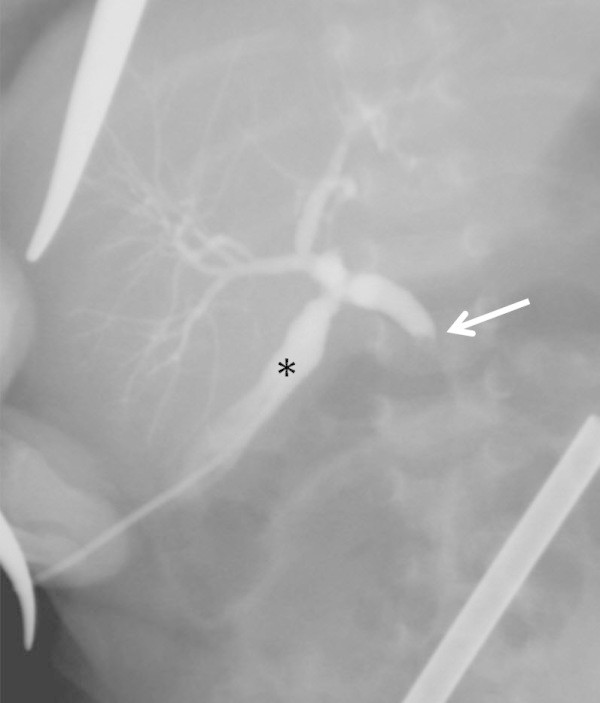
**Intraoperative cholangiography via the punctured gall bladder (asterisk) showing complete obstruction of common bile duct (arrow) with dilatation of cystic duct and hepatic duct.**

**Figure 2:**
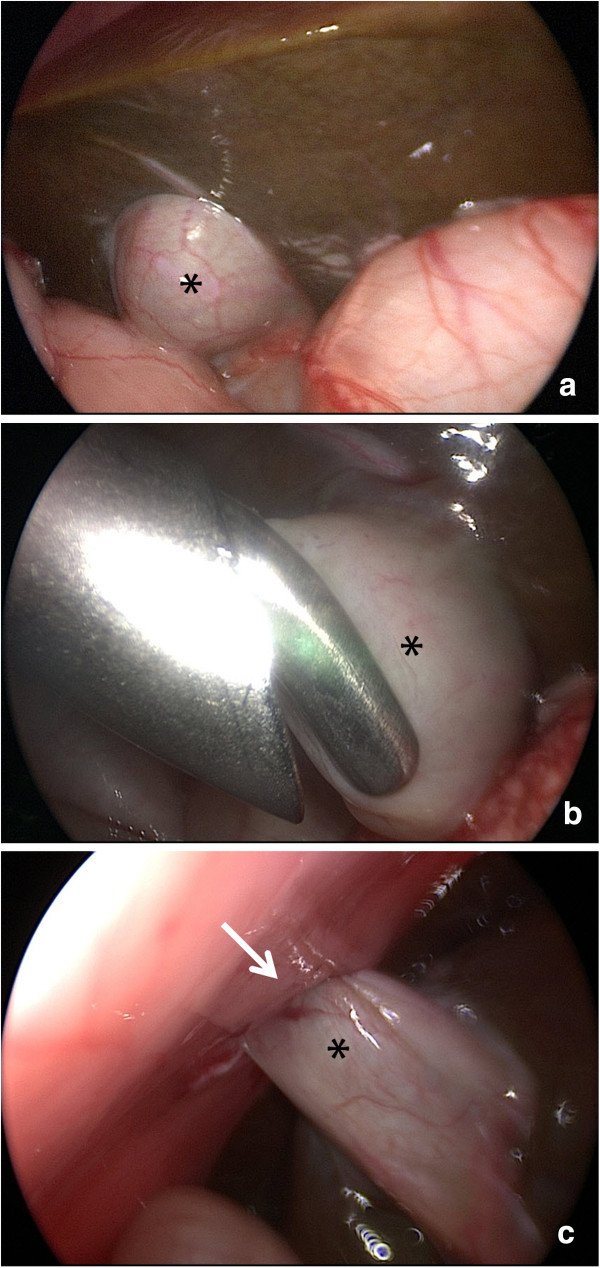
**Intraoperative laparoscopic views of the gall bladder (asterisk) and liver surface (a), the gall bladder being grasped and pulled to the trocar (b), after suturing the gall bladder to the fascia (c), arrow marking the trocar site.**

**Figure 3:**
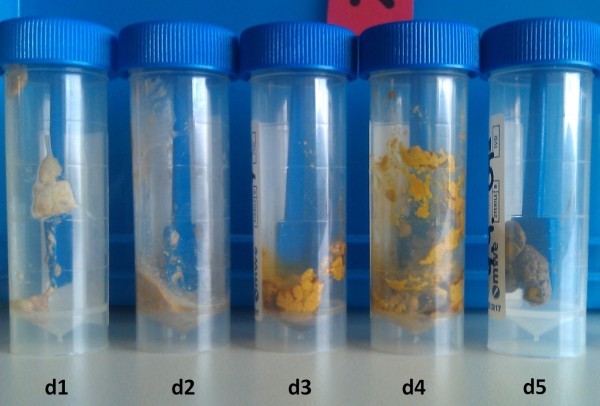
**Postoperative course of stool pigmentation.**

**Figure 4:**
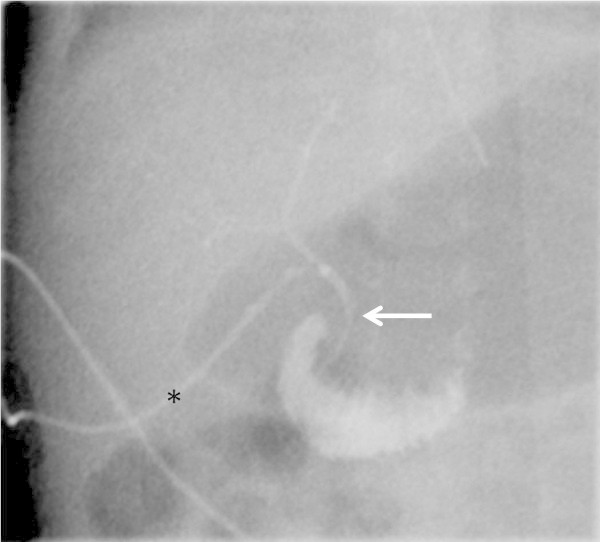
**Contrast study via the gall bladder catheter after 8 days of irrigation showing narrow bile ducts and free passage into the duodenum, arrow marking the common bile duct, asterisk marking the catheter placed into the gall bladder.**

The case of a 6 weeks old boy with inspissated bile syndrome following prolonged intravenous antibiotics and parenteral nutrition after complicated repair of esophageal atresia is reported. Inspissated bile syndrome was diagnosed when sludge was found in the gall bladder and choledochal duct sonographically and hyperbilirubinemia (50 μmol/l), elevation of liver enzymes (gammaglutamatetransferase 447 U/L, alcalic phosphatase 373 U/L) and feeding intolerance developed. Cholestasis was unresponsive to an oral trial of ursodesoxycholic acid for 14 days. After laparoscopically controlled (umbilical trocar, 3 mm) transabdominal puncture of the gall bladder, intraoperative cholangiography revealed complete obstruction of the common bile duct (Figure 1). Therefore, the gall bladder was grasped through a second 3 mm port in the right upper abdomen and pulled out at the port site, sutured to the fascia extracorporally, and an indwelling 4 French Certofix catheter was positioned into the infundibulum (Figure 2). At the fascia level a purse string suture was knotted around the catheter and the skin was closed with 3 stiches above. The catheter was used for daily sterile irrigation with ursodesoxycholic acid. After 3 days, pigmentation of the stool was observed (Figure 3) and a contrast study via the catheter at day 8 revealed complete resolution of the plug and free passage of contrast medium into the duodenum (Figure 4). The catheter was removed on day 8, skin closure was observed 2 days later. Upon sonographic and laboratory follow up at 3 months normal extra- and intrahepatic bile ducts were found, no signs of cholestasis were detected. This technique appears simple, effective and avoids a second surgery since the catheter is only removed after resolution of the bile obstruction without the need for closure of the gall bladder.

### Review of surgical options

The probability for the need for a surgical intervention is high when bile ducts become dilated to more than 3 mm (Fitzpatrick et al. 2010). Bile duct dilatation, persisting jaundice and increasing laboratomy parameters such as alcalic phosphatase, ASAT, ALAT, and y-GT are regarded as indication for intervention. Besides the possibility for irrigation of the bile ducts, laparoscopy and intraoperative cholangiography are suitable to rule out biliary tract malformations as a reason for cholestasis when combined with a liver biopsy. Several procedures are described for clearance of obstructed bile ducts: percutaneous management under sedation with transhepatic puncture of the gall bladder and introduction of a wire and a balloon dilator has recently been reported (Duman et al. 2011). The majority of authors describe laparoscopic aided cholecystostomy under anesthesia with placement of a catheter into the gall bladder for repeated irrigation with saline (Gao et al. 2011) or ursodesoxycholic acid (Gunnarsdottir et al. 2008; Lieber et al. 2012). Identification of the correct position of the port for pulling out the gall bladder as described by Gao et al. ( 2011) is crucial. We performed daily lavage of the gall bladder with boluses of 5 ml saline and then installed 3 ml of ursodesoxycholic acid (50 mg/ml or 50 mg/kg body weight). Sonographic controls were withheld until the stool became coloured and hyperbilirubinemia resolved. Prior to pulling the catheter, a contrast radiological study is recommended to record that complete clearance of the bile ducts has been achieved. It is not clear however, whether a second procedure is necessary after removal of the catheter. The need for gall bladder closure and detachment from the abdominal wall may depend on the type of catheter employed. With the use of a balloon catheter, closure of the gall bladder has been reported after its removal to maintain gall bladder function and to avoid adhesions (Lieber et al. 2012). We used a 4 F (16 G) central venous catheter (Certofix Mono S 415, B. Braun, Melsungen, Germany) which appeared to us ideal in length (15 cm), rigidity and its features for fixation to the skin. The smaller diameter of this catheter allowed for rapid spontaneous closure of the cholecystostomy opening after catheter removal. In the present case the cholecystostomy site was dry after 24 hours and only a minimal scar was visible at follow up after 3 months. There is no long term follow up available for both techniques however.

## Conclusions

A one stage minimally invasive procedure was efficient and safe for clearance of a completely obstructed common bile duct and gall bladder in inspissated bile syndrome in an infant.
